# Radiofrequency Ablation of the Costotransverse Joint in a Case of Chronic Thoracic Back Pain

**DOI:** 10.7759/cureus.63958

**Published:** 2024-07-06

**Authors:** Janus Patel, Ashley M Eaves, Emily Deschler, Christian Bowlin, Enrique Galang

**Affiliations:** 1 Department of Anesthesiology/Comprehensive Pain and Spine Medicine, Wake Forest University School of Medicine, Winston-Salem, USA; 2 Department of Physical Medicine and Rehabilitation, Vanderbilt University Medical Center, Nashville, USA

**Keywords:** costotransverse joint radiofrequency ablation, costotransverse joint rfa, thoracic facet joint, thoracic imaging, facet joints injection, symptomatic macromastia, costotransverse joint pain, radiofrequency ablation (rfa), thoracic back pain, costotransverse joint

## Abstract

The source of thoracic back pain is often challenging to diagnose and manage, as there exist multiple potential etiologies and treatment strategies. Costotransverse joints are small synovial joints that may be prevalent and overlooked pain generators in the thoracic spine. Intra-articular steroid injections are commonly utilized as non-surgical therapeutic interventions for costotransverse joint pain; however, they have variable efficacy. We describe the first use of thermal radiofrequency ablation for the symptomatic management of chronic thoracic back pain in a 35-year-old female with costotransverse joint arthropathy. The patient presented with thoracic hypomobility, severe pressure sensation, and dull pain in the T7-10 region bilaterally between the medial border of the scapulas. Initial treatment with physical therapy, pain medications, and a thoracic epidural steroid injection were ineffective. Computed tomography thoracic spine imaging revealed isolated arthropathy of the costotransverse joints at T8 and T9 bilaterally. Initial treatment with an intra-articular steroid injection provided significant short-term pain relief and was followed by a diagnostic block, which resulted in over 80% pain relief. Thereafter, thermal radiofrequency ablation of the nerves to the costotransverse joints at T8 and T9 was performed. The patient experienced three months of pain relief, resulting in functional improvement and reduced pain medication requirements. This case underscores the importance of considering costotransverse joint pathology in the differential diagnosis of thoracic back pain, the critical role of radiographic imaging in establishing prevalence, and the need for further anatomic studies describing the complete innervation of the costotransverse joints to optimize thermocoagulation treatments.

## Introduction

Chronic back pain is a leading source of disability worldwide, affecting approximately 66% of the general population, with 15% of patients reporting pain in the thoracic region [1]. Thoracic back pain without myeloradiculopathy often proves difficult to treat due to multiple potential overlapping pain etiologies in the thoracic spine, such as degenerative discs, joint (zygapophyseal facet, costovertebral joint (CVJ), and costotransverse joint (CTJ)) degeneration, and myofascial pain. Manchikanti et al. reported a 42% prevalence of facet joint pain in patients with chronic thoracic spine pain [2]. Radiofrequency ablation (RFA) of the medial branches of the dorsal ramus of the corresponding target level, as well as the level above, is commonly performed to denervate the zygapophyseal facet joints, thereby reducing back pain emanating from these specific facet joints. Unique to the thoracic spine, there are two additional synovial joints that are overlooked pain generators, namely the CVJs and CTJs. CTJs are formed by the articulation of the tubercle of the posterior rib with the corresponding transverse process of the thoracic vertebra, present at all thoracic levels except T11 and T12 [3]. The CVJs and CTJs, along with their associated ligaments, play a crucial role in thoracic stabilization, mobility, and respiratory function to allow for maximum expansion of the chest wall [4].

The historical belief that CTJs could not transmit pain has been refuted, as somatic and autonomic neuropeptides have been identified in the CTJ capsule [3]. CTJ-mediated pain has been commonly described as a deep, dull ache and pressure sensation [5]. Currently, there is limited epidemiological data describing the prevalence of and causative mechanisms for developing non-rheumatologic CTJ pathology. The innervation of the CTJs is thought to arise from articular branches emanating from the lateral branch of the posterior rami of the corresponding level [6]. However, there is a paucity of anatomical data describing the full innervation and most common nerve locations of the CTJs, which often serve as a guide for interventional procedures such as RFA. To our knowledge, non-surgical interventional treatment for CTJ-mediated pain has been limited to intra-articular steroid injections. Herein, we describe the use of RFA for the treatment of isolated CTJ-mediated chronic thoracic back pain.

## Case presentation

A 35-year-old female with no pertinent past medical history presented to our outpatient interventional pain medicine clinic for evaluation of chronic thoracic back pain without radiculopathy. Conservative management with physical therapy, heat therapy, chiropractic care, analgesics, opioids, and muscle relaxant medications failed to provide adequate pain relief as the patient experienced severe pain rated 8-10/10, which was further aggravated by hyperextension, rotation, and lateral bending. The pain was generalized in a non-distinct area encompassing the T7-T10 region bilaterally between the lower medial scapula borders. The patient described the pain as dull, achy, and with a sharp pressure sensation.

**Figure 1:**
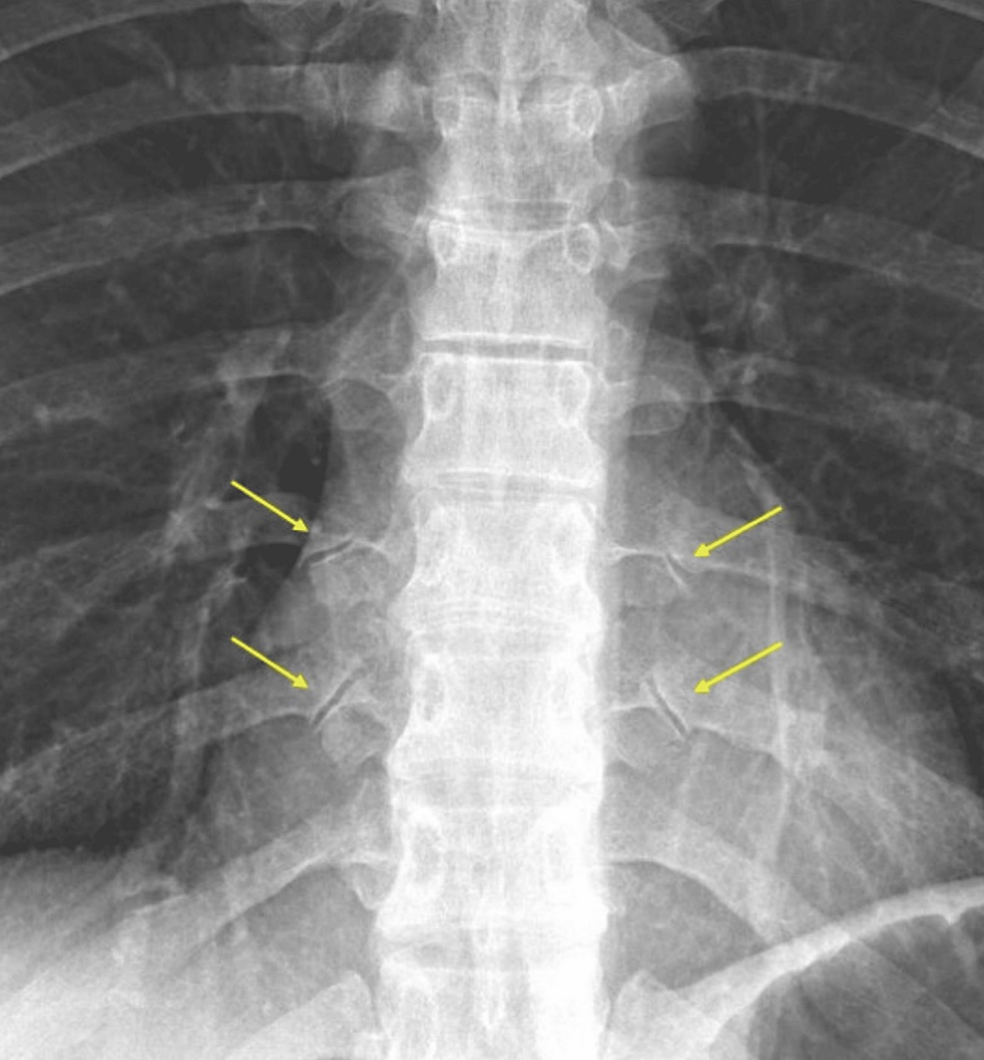
X-ray of the thoracic spine showing degenerative changes in the bilateral T8 and T9 costotransverse joints (yellow arrows).

**Figure 2:**
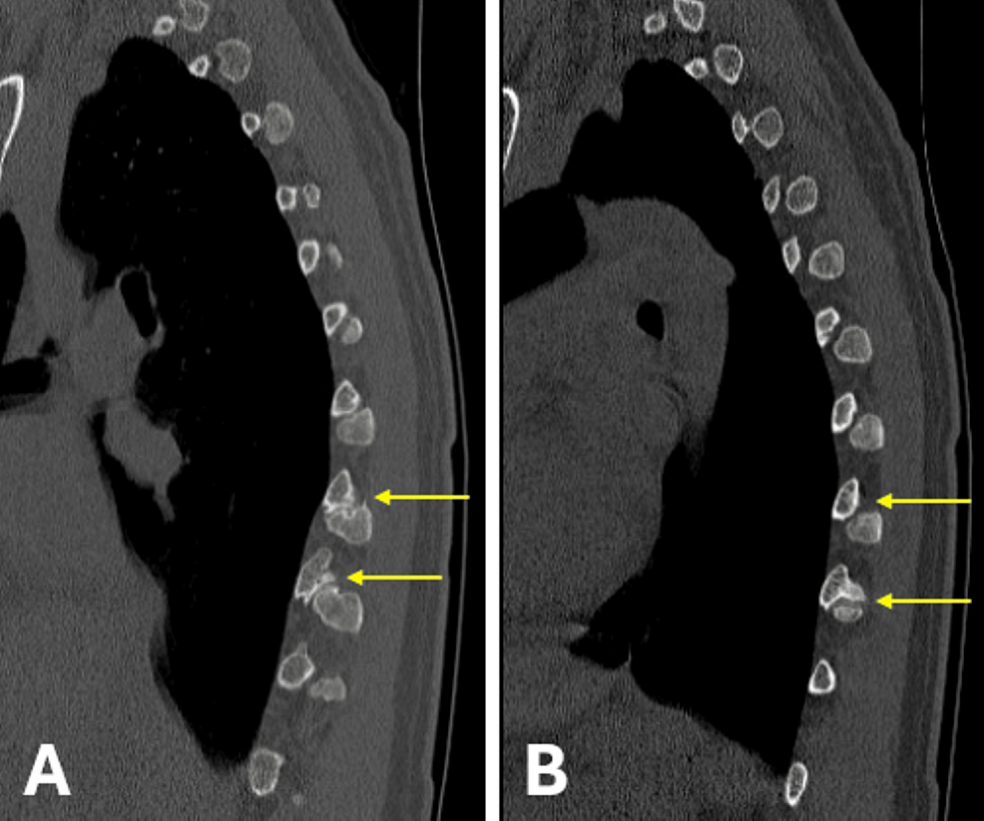
Sagittal CT scan images of the thoracic spine illustrating arthropathy of the costotransverse joints at the T8 and T9 levels bilaterally (yellow arrows). (A) Right sagittal CT scan; (B) left sagittal CT scan. CT: computed tomography

**Figure 3:**
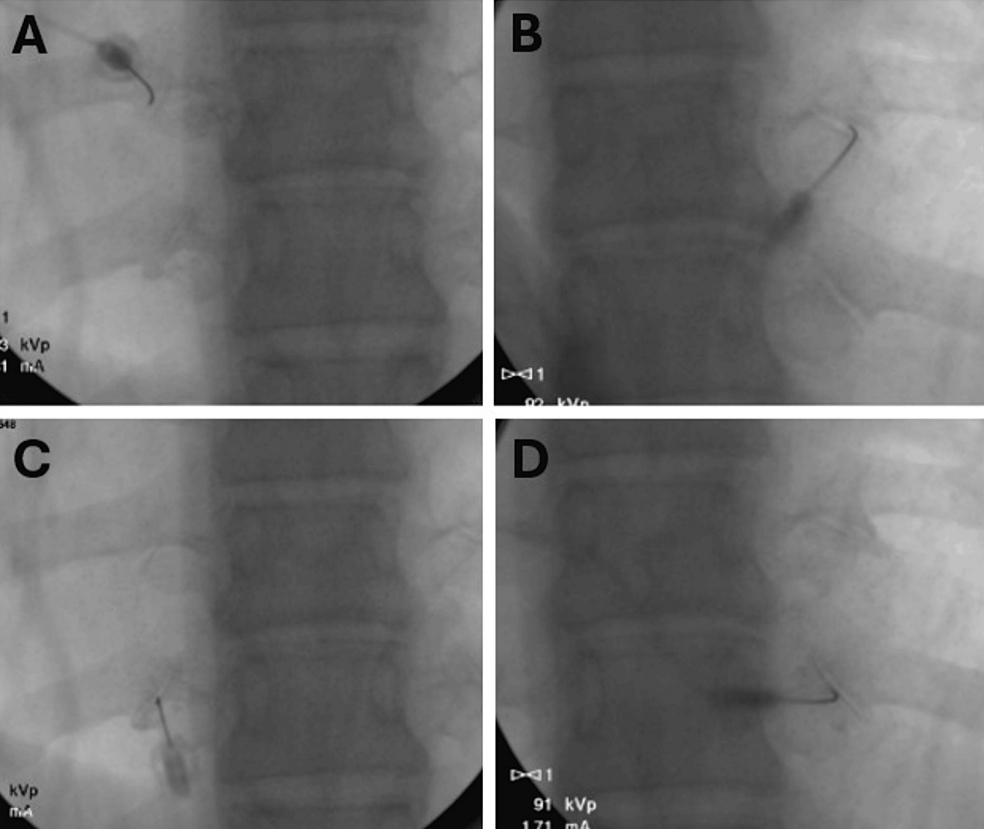
Fluoroscopic images showing needle placement at the (A) left T8 CTJ line, (B) right T8 CTJ line, (C) left T9 CTJ line, and (D) right T9 CTJ line for diagnostic and therapeutic intra-articular steroid injections. CTJ: costotransverse joint

The physical exam was positive for tenderness along the T7-T10 thoracic paraspinal muscles bilaterally. Initial treatment with a thoracic interlaminar epidural steroid injection at T6-T7 failed to provide meaningful and durable pain relief. Plain films of the thoracic spine showed obvious degenerative changes in the CTJs (Figure 1). Magnetic resonance imaging (MRI) of the thoracic spine was negative for degenerative discs and neuroforaminal and central canal stenosis. A computed tomography (CT) scan of the thoracic spine was obtained and confirmed moderate degenerative changes at the bilateral T8 and T9 CTJs with normal zygapophyseal facet joints and CVJs (Figure 2). Supported by history, physical exam findings, radiographic imaging, and exclusion of other pathology to explain symptoms, we suspected the CTJs to be the primary source of pain. Initial treatment with intra-articular steroid injections of the CTJs yielded significant short-term relief, further supporting a CTJ-mediated pain etiology (Figure 3).

Fluoroscopic-guided diagnostic blocks of the bilateral T8 and T9 nerves to the CTJs

Given the lack of provocative physical examination maneuvers to recreate CTJ-mediated thoracic pain, as well as the lack of detailed location-specific anatomic description of CTJ innervation, a double-blinded method was employed for the diagnostic blocks, such that the patient and examiner were both blinded to the injectate. The importance of laterality was also tested.

**Figure 4:**
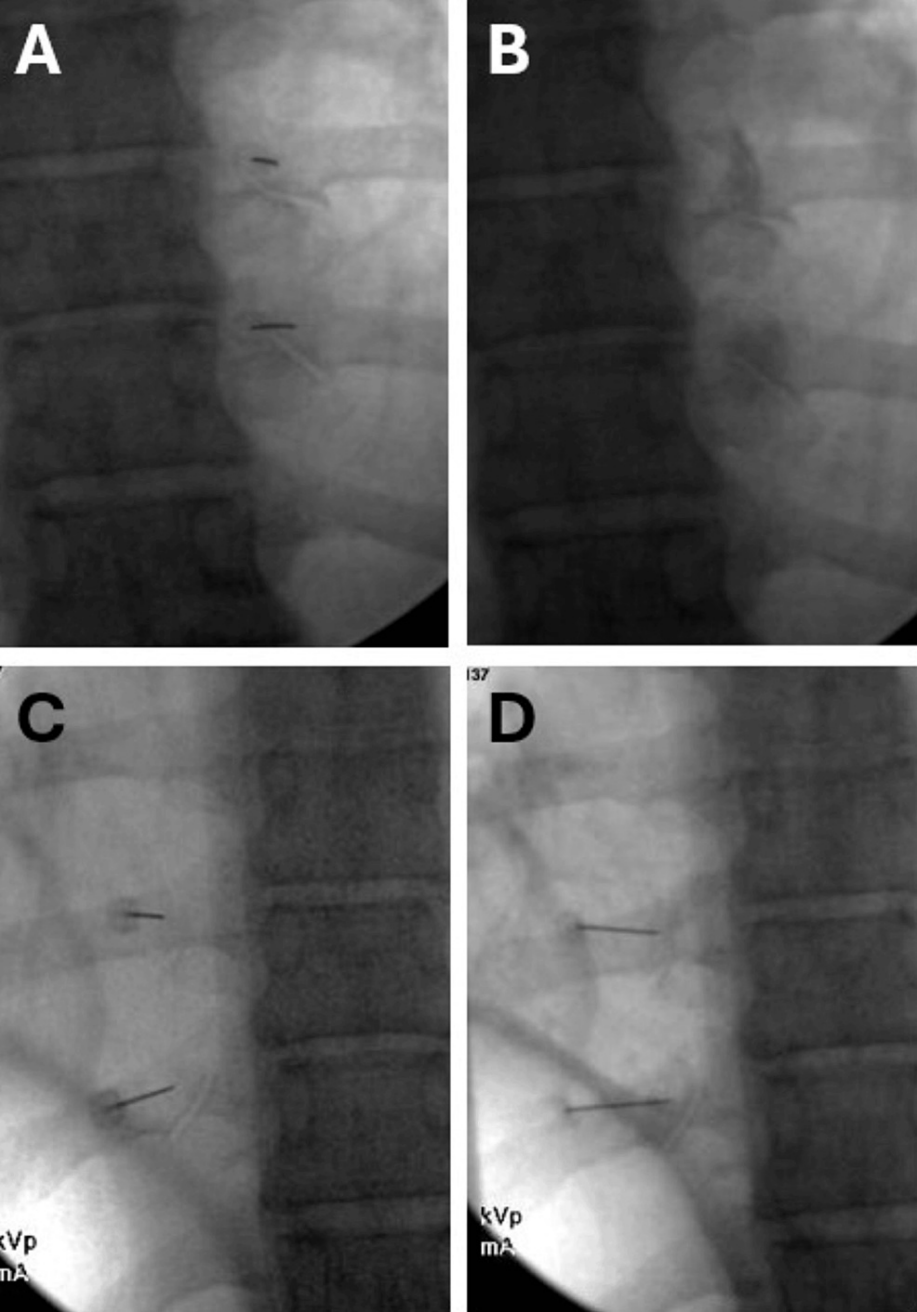
Fluoroscopic images showing needle placement for the purely diagnostic CTJ nerve blocks, targeting the superior and lateral areas to the superolateral corner of the transverse process at the T8 and T9 levels bilaterally. (A) The needles were placed at the right T8 and T9 CTJs for the first injection. (B) Spread of iodinated contrast on the right side following the first injection. (C) The needles were placed at the left T8 and T9 CTJs for the second injection. (D) Spread of iodinated contrast on the left side following the second injection CTJ: costotransverse joint

Fluoroscopic imaging of the T8 and T9 CTJs was obtained as previously described. A target zone approximately superior and lateral to the superolateral corner of the target level transverse process was chosen based on limited available literature describing potential locations of nerve innervation to the CTJ [6,7]. A 25-gauge, 3.5-in curved spinal needle was advanced until bony contact was made within the targeted area. Needles were placed bilaterally at all four target sites at T8 and T9 (Figures 4A, 4C). Then, the right side was injected with 0.5 mL of a mixture consisting of a 1:1 ratio of 4% lidocaine and iodinated contrast (Figure 4b), while the left side was injected with 0.5 mL of normal saline. All needles were removed, and the blinded examiner evaluated the patient and performed data collection consisting of pain scores and exam maneuver results. Then, the patient was placed back on the procedure table, and sterile conditions were again obtained. Needle placement of all four target sites at bilateral T8 and T9 was performed as described above. After bony contact along the intended target zones was obtained, the left side was injected with 0.5 mL of the same 1:1 ratio of 4% lidocaine and contrast solution (Figure 4D), while the right side was injected with 0.5 mL of normal saline. The needles were then removed, and the patient was re-examined by the blinded examiner.

In order to assess post-procedure pain and functional improvement, we devised two exam maneuvers, the “Almost Apprehension Test” and the “Standing Y Raise Test." The Almost Apprehension Test was adapted from the Apprehension Test for shoulder instability and involves recording the duration of time that the patient can keep their arms raised at 90° of shoulder abduction and 90° of elbow flexion. The Standing Y Raise Test involves recording the duration of time that the patient can keep both arms raised above the head in the Y position with elbows fully extended. The patient showed notable improvement in performance times for the Almost Apprehension Test and the Standing Y Raise Test following the first and second blocks, with the longest performance times demonstrated with the final exam maneuvers at 15 minutes after the first block (10 minutes after the second block). Following both blocks, complete resolution of bilateral pressure pain in the thoracic region was achieved (Table 1). In addition, the sharp pain was eliminated on the right side and drastically reduced on the left side. Because the patient reported over 80% relief of thoracic back pain, we moved forward with bilateral RFAs of the nerves to the T8 and T9 CTJs.

**Table 1:** Pain scores for pressure and sharp pain and exam maneuver performance times at baseline and following diagnostic blocks of the bilateral T8 and T9 nerves to the CTJ. The first block treated the right T8 and T9 CTJs. The second block treated the left T8 and T9 CTJs. CTJ: costotransverse joint

	Pre-procedure (Baseline)	5 Minutes Post-block #1	10 Minutes Post-block #1 (5 Minutes Post-block #2)	15 Minutes Post-block #1 (10 Minutes Post-block #2)
Left-sided pressure pain score	10/10	0/10	0/10	0/10
Right-sided pressure pain score	8/10	0/10	0/10	0/10
Left-sided sharp pain score	7/10	2/10	2/10	2/10
Right-sided sharp pain score	7/10	0/10	0/10	0/10
Almost Apprehension Test time	15 seconds	22 seconds	48 seconds	51 seconds
Standing Y Raise Test time	51 seconds	83 seconds	86 seconds	253 seconds

Thermal RFAs of the bilateral T8 and T9 nerves to the CTJs

**Figure 5:**
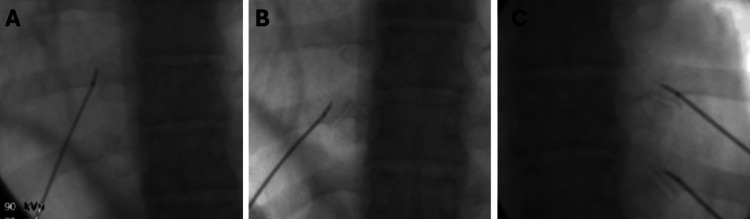
Fluoroscopic images showing cannula placement for thermal radiofrequency ablation of the bilateral T8 and T9 nerves to the CTJ. (A) PA fluoroscopic image showing cannula placement targeting the left T8 CTJ; (B) PA fluoroscopic image showing RF cannula placement targeting the left T9 CTJ; (C) PA fluoroscopic image showing RF cannula placement targeting the right T8 and T9 CTJs CTJ: costotransverse joint; PA: posteroanterior; RF: radiofrequency

Posteroanterior (PA) fluoroscopic imaging of the right T8 and T9 CTJs were obtained as previously described. The target zone for ablation was chosen along the superior CTJ joint border at the angle of the joint line. An 18-gauge 100-mm radiofrequency cannula with a 10-mm curved active tip was advanced using intermittent fluoroscopy until bony contact was made along the superolateral aspect of the CTJ. Then, sensory stimulation was conducted until a point at which the patient felt a “pressure” sensation in an area inferior and lateral to the needle entry site. After appropriate sensory testing, 1 mL of 2% lidocaine was administered, and RFA was performed at 80 °C for 90 seconds (Figure 5).

Three weeks following RFA, the patient reported pain reduction from 10/10 to 5-6/10 on average. In addition, the patient demonstrated functional improvement, achieved a more active lifestyle without using heating pads, and discontinued tramadol. Although the patient endorsed 40%-60% relief in pain following the RFA, the quality of pain remained unchanged. Nonetheless, RFA was successful and provided this patient with approximately three months of thoracic back pain relief before the return of symptoms.

## Discussion

This is a description of RFA for treating chronic thoracic back pain from isolated CTJ degeneration, with subsequent functional improvement and a decrease in medication requirements for approximately three months before the return of symptoms. CTJs play an important role in chest wall expansion and respiratory mechanics, supporting the ability of the chest wall to move “up and out” during respiration [4]. Recent studies in patients with chronic obstructive pulmonary disease (COPD) revealed a significantly higher prevalence of CTJ arthropathy in patients with COPD and thoracic back pain [8,9]. In addition, a recent study of inflammation of the CTJs in a small sample of symptomatic patients noted a higher prevalence of CTJ pain in females than in males, suggesting that CTJ pathology may be linked to female morphology [10]. Given the lack of rheumatologic, infectious, or other potential etiologies leading to the development of CTJ arthropathy in our case, we hypothesize that CTJ degeneration in our patient may be due to macromastia. Repetitive CTJ “up and out” motion during respiration coupled against the restrictive forces of chronic forward flexion and decreased chest wall compliance from hypertrophic dense breast tissue may ultimately play a role in the development of CTJ degeneration over time [11]. Future epidemiologic investigations examining the prevalence of CTJ pathology and thoracic back pain in the general population, as well as the female population desiring reduction mammoplasty for macromastia and thoracic back pain, are warranted.

The ability of most clinicians to recognize CTJ degeneration radiographically is an area for clinical improvement. In our case, a simple anteroposterior radiograph of the thoracic spine was sufficient to screen for CTJ degeneration as the affected joints showed prominent, irregular joint lines and were far lateral from the midline structures. Radiographic images were used as a screening tool, which prompted further confirmatory imaging studies with CT imaging of the thoracic spine. MRI imaging of the thoracic spine did not adequately capture the nature of degenerative bony change in the CTJ, and radiology reports of both the thoracic radiographs and MRI failed to mention the presence of CTJ pathology. Thus, scrutiny of thoracic radiographs and any available CT imaging is paramount in the workup for thoracic back pain.

There are several limitations and future areas of research that may better guide interventional techniques and optimize the duration and amount of clinical improvement from CTJ RFA. First, scant cadaveric anatomic literature exists that specifically describes sensory innervation to the CTJ along with anatomic location using bony landmarks. Given the complexity of thoracic back pain and the multitude of potential pain sources, future anatomic characterization of CTJ sensory innervation will be vital for optimal pain control using RFA. Three months of clinical improvement observed in our case may be due to partial thermocoagulation, as articular branches may innervate various portions of the CTJ and may not have been within our target thermocoagulation zone. Second, the target nerve's location may not be immediately adjacent to a bony structure, such as the transverse process or rib. The articular branches of the CTJ, which arise from the lateral branch of the posterior rami, may be located and better targeted within the intertransverse space. However, targeting thermocoagulation at the intertransverse location carries a higher risk of inadvertent pneumothorax if performed under fluoroscopic guidance only. Lastly, our use of a traditional radiofrequency cannula with a 10-mm active tip provided a limited surface area of thermocoagulation. Future RFA intervention of the CTJ using a bipolar technique, cooled radiofrequency, or bifurcated-tine radiofrequency cannula may be better suited for increasing the total surface area of thermocoagulation.

## Conclusions

CTJ arthropathy may be a highly prevalent but overlooked and underdiagnosed etiology of thoracic back pain. This case highlights the importance of considering CTJ pathology in the diagnostic workup for patients experiencing thoracic back pain. We described a novel interventional approach for treating thoracic back pain associated with CTJ arthropathy by targeting the innervation of the CTJs with diagnostic nerve blocks followed by thermal RFA, which provided only three months of pain relief and functional improvement in this case. Cadaveric anatomic description of CTJ innervation and identification of bony fluoroscopic landmarks are areas of future research. RFA may be an effective, minimally invasive treatment option for CTJ arthropathy that is refractory to conservative management.
